# Efficacy and Safety of a Stimulator Using Low-Intensity Pulsed Ultrasound Combined with Transcutaneous Electrical Nerve Stimulation in Patients with Painful Knee Osteoarthritis

**DOI:** 10.1155/2019/7964897

**Published:** 2019-06-16

**Authors:** Eu-Deum Kim, Yu Hui Won, Sung-Hee Park, Jeon-Hwan Seo, Da-Sol Kim, Myoung-Hwan Ko, Gi-Wook Kim

**Affiliations:** ^1^Department of Physical Medicine & Rehabilitation, Chonbuk National University Medical School, Jeonju 54907, Republic of Korea; ^2^Research Institute of Clinical Medicine of Chonbuk National University—Biomedical Research Institute of Chonbuk National University Hospital, Jeonju 54907, Republic of Korea; ^3^Translational Research & Clinical Trial Center for Medical Device, Chonbuk National University Hospital, Jeonju 54907, Republic of Korea

## Abstract

**Objective:**

Studies regarding the combination of ultrasound and transcutaneous electrical nerve stimulation (TENS) are rarely reported. In this study, we aimed to elucidate the efficacy and safety of a stimulator using low-intensity pulsed ultrasound (LIPUS) combined with TENS in patients with painful knee osteoarthritis (OA). We evaluated the effectiveness of this therapy against pain, physical function, and cartilage regeneration. Moreover, we aim to prove the superiority of the effects of LIPUS combined with TENS therapy compared with only TENS therapy.

**Methods:**

Of the 40 included patients, aged 45–85 years with painful knee OA, 20 patients received only TENS therapy and 20 patients received LIPUS combined with TENS therapy for 8 weeks (a total of more than 80 treatment sessions). We evaluated visual analogue scale (VAS), Western Ontario and McMaster Universities (WOMAC) osteoarthritis index, MOS 36-Item Short-Form Health Survey (SF-36), and femoral articular cartilage (FAC) thickness. The evaluation was performed at three visits: visit 1 (V1, pretreatment, within 28 days after screening), visit 2 (V2, posttreatment period 1, ±3 days after treatment), and visit 3 (V3, posttreatment period 2, 21 ± 3 days after treatment).

**Results:**

We expected that LIPUS combined with TENS therapy would be superior to only TENS therapy. However, there was no significant difference between the two therapies. In the within-group comparison, both treatments (only TENS therapy and LIPUS with TENS therapy) demonstrated statistical differences from baseline values for pain and physical function outcomes. FAC thickness showed no significant differences after treatment in both groups.

**Conclusion:**

The effects of a stimulator using LIPUS with TENS on pain relief and functional improvement were not superior to the only TENS therapy. Cartilage regeneration, which was expected as an additional benefit of LIPUS, was also not significantly evident. Therefore, further investigation is warranted to determine whether the combination therapy is beneficial. This trial is registered with KCT0003883.

## 1. Introduction

Knee pain is a common musculoskeletal symptom in elderly populations, and its prevalence increases with age [1]. In a previous study, 28.3% of adults aged more than 40 years reported that they experienced knee pain on most days for at least 1 month [2]. According to the Korea National Health and Nutrition Examination Survey, which was conducted from 2010 to 2012, the prevalence of knee pain is 20.7% in adults aged over 50 years [3]. The possible causes of knee pain are osteoarthritis (OA), gout, popliteal cyst, patellofemoral pain syndrome, ligament or tendon injury caused by trauma, and septic arthritis [4]. In another study, 16% of the African American or Caucasian population aged 45 years and older complained of knee pain and had higher than Kellgren and Lawrence (K-L) grade 1, which is defined as radiologic knee OA [5]. Thus, knee OA is one of the most common causes of knee pain, and it affects physical function and quality of life and can lead to psychological distress [6, 7].

According to the American College of Rheumatology and Osteoarthritis Research Society International, recommendations for the nonsurgical treatment of knee OA include exercise, weight loss, biomechanical interventions, acetaminophen, oral or topical nonsteroidal anti-inflammatory drugs (NSAIDs), and intra-articular corticosteroid injection [8, 9].

Transcutaneous electrical nerve stimulation (TENS) is a physical modality that has been widely used to relieve pain in patients with knee OA [10]. Although several types of electrical stimulation are available, conventional TENS is the most commonly applied [11]. Therapeutic ultrasound is also a frequently used modality for the treatment of knee OA-associated pain. Moreover, there are some studies about the effects of low-intensity pulsed ultrasound (LIPUS) on cartilage repair in patients with knee OA [12].

The effects of TENS or therapeutic ultrasound in knee OA have been widely investigated. A combination of physical modalities, such as TENS, therapeutic ultrasound, and high-intensity laser treatment, has been also reported [10, 13, 14]. However, only few studies are available about the efficacy and safety of ultrasound combined with TENS (ultraTENS) machine, which simultaneously generates an ultrasound wave and electrical stimulation, in knee OA [15]. They only investigated about pain and functional outcomes in patients with knee OA. Cartilage regeneration, which could be an additional benefit of low-intensity ultrasound, was not examined. No data were also available about long-term follow-up.

Therefore, in this prospective, randomized, single-blind (assessor), comparative controlled trial, we aimed to elucidate the efficacy and safety of a stimulator using LIPUS combined with TENS in patients with painful knee OA. We evaluated the effectiveness of this therapy against pain, physical function, and cartilage regeneration in these patients. Moreover, we aim to prove the superiority of the effects of LIPUS combined with TENS therapy compared with only TENS therapy, which is widely used in clinical field.

## 2. Materials and Methods

### 2.1. Participants

This study was approved by the Institutional Review Board of Chonbuk National University Hospital (Approval number: CUH 2017-08-005). Study subjects were recruited through a notice posted on the bulletin board of the hospital and screened by a rehabilitation physician. The participants were explained the details of the study prior to obtaining written informed consent. The study was performed in accordance with the Declaration of Helsinki. The inclusion criteria were patients aged over 18 years with knee pain. All participants who were classified as K-L grade I to IV by standing posteroanterior X-ray in 15° knee flexion were eligible for this study. Exclusion criteria included any patient with a history of knee surgery within the last 6 months, history of steroid injection or surgery in the lower extremity within the last 1 month, knee joint infection, inflammatory joint disease, acute tendon or ligament injury of the knee, dementia or cognitive impairment, neurological disorders such as central nerve system disorder, lumbosacral radiculopathy or polyneuropathy, and hypesthesia in the lower extremity, and pregnant women. All participants were assessed by one investigator who was a board-certified physiatrist and blinded to group allocation. This study is registered at the Clinical Research Information Service, which is conducted by the Korea Centers for Disease Control and Prevention (Registration number: KCT0003883).

### 2.2. Study Design

This study was a single center, prospective, randomized single-blind (assessor), comparative controlled trial. Each subject who fulfilled the inclusion criteria randomly was allocated to the LIPUS combined with TENS group or the only TENS group by simple-computerized random number generator. A clinical research coordinator who was a clinical research nurse and not involved in the assessments was responsible for allocating the participants. Therefore, group allocation was concealed to all of the investigators. The affected knee was assessed. When both knees from the same participant were eligible, we included the more painful knee.

Baseline data included the standing posteroanterior X-ray in 15° knee flexion and K-L scale, which was determined by two rehabilitation physicians and one radiologist. The other baseline data were the physical examination of the knee, vital signs, age, medical history, and urine tests for pregnancy. A clinical research coordinator educated patients on how to manipulate the TENS machine or a stimulator using LIPUS combined with TENS. Each patient took a device (TENS machine or a stimulator using LIPUS combined with TENS) home and administered home-based self-therapy. Both groups underwent a 20-minute self-therapy per session, which was performed 3 or less than 3 sessions per day and more than 10 sessions per week for 8 weeks. Thus, the total treatment session was more than 80 sessions. They completed a self-therapy checklist daily. A clinical research coordinator contacted the patients by telephone once a week and visited at home once a month to monitor the home-based self-therapy. Participants were only allowed to take their pain medication which was started at least two months before the screening. They were not allowed to change the dose or type of pain medication or start any other types of treatments for knee OA during the trial. In addition, participants were requested not to change their physical exercise level.

**Figure 1:**
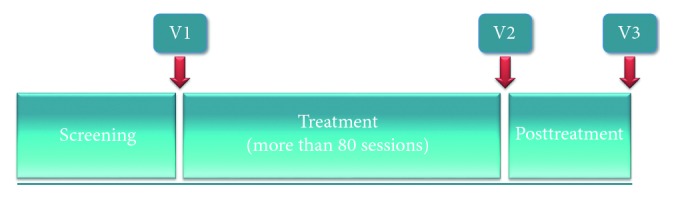
Experimental design.

Knee pain, function, and thickness of femoral articular cartilage (FAC) of the participants were evaluated. The evaluation was performed at three visits: visit 1 (V1, pretreatment, within 28 days after screening), visit 2 (V2, posttreatment period 1, ±3 days after treatment), and visit 3 (V3, posttreatment period 2, 21 ± 3 days after treatment) (Figure 1). All evaluations were performed by the same investigator who was a board-certified physiatrist and blinded to group allocation.

### 2.3. Interventions

#### 2.3.1. Only TENS Therapy

A commercially available TENS machine (Chil-Sung, Co, Ltd., South Korea) was used for stimulation. The TENS setting was in a conventional mode, with a frequency of 100 Hz and a pulse duration of 50–100 *μ*s. The participant was placed in a sitting position with the affected knee flexed at 90°. Two 5 × 5 cm electrodes were placed above the patella, and 2 were placed below. The intensity of the stimulation was set to low intensity to stimulate large diameter, low threshold non-noxious afferent fibers (A-beta). Thus, the stimulation intensity was set to produce a strong tingling sensation, but without pain [16].

#### 2.3.2. LIPUS Combined with TENS Therapy

LIPUS combined with TENS therapy was performed using CARESTAR (GENEMEDI Co, Ltd., South Korea). CARESTAR consists of two 2.8 cm diameter applicators and gives LIPUS energy and TENS in 1-s shifts. Therefore, 50% of the stimulation was offered by LIPUS, and remaining 50% was provided by TENS. The LIPUS signal is transmitted at a frequency of 1 MHz, with an intensity of 0.1 W/cm^2^. The effective radiating area was 3.3 cm^2^. The duty cycle of pulsed ultrasonic waves was 40%. The TENS setting was in a conventional mode, with a frequency of 80 Hz and a pulse duration of 50–100 *μ*s. The intensity of TENS current was set to produce a strong tingling sensation, but without pain. The participant was placed in a sitting position, with the affected knee flexed at 90° to enhance ultrasonic energy penetration into the joint space [17]. A nondrug coupling gel was applied. The participant was taught to allocate the two applicators medial and lateral to the involved knee by fixing with an elastic band.

### 2.4. Outcome Measures

The primary outcome was knee pain measured using a visual analogue scale (VAS). On a 100 mm long line, point 0 indicates no pain and point 10 indicates the most severe pain. The participants marked the intensity of their knee pain on this line. The distance between point 0 and the point that the participants marked was measured with a ruler [18]. We evaluated knee pain by using VAS in three different conditions. VAS-P1 was regarded as pain at the current moment, VAS-P2 as pain with knee movement, and VAS-P3 as pain at resting position.

Secondary outcomes were evaluated with Western Ontario and McMaster Universities (WOMAC) osteoarthritis index, MOS 36-Item Short-Form Health Survey (SF-36), and FAC thickness.

The WOMAC index is widely used to evaluate pain, stiffness, and physical function in patients with knee or hip OA. It consists of 24 items, and higher scores represent worse pain, stiffness and impaired physical function [19].

The SF-36 is a self-administered questionnaire containing 36 items that survey overall health status. It measures health on eight multi-item dimensions, covering physical functioning, physical role limitation, emotional role limitations, energy/vitality, mental health, social functioning, pain, and general health perceptions. Precoded numeric values are recoded as per the scoring from 0 to 100. Therefore, each item is scored on a 0–100 range. In the same dimension, the scores of the items are averaged together. The percentage scores of all the eight dimensions were summed and divided by 8 to arrive at a global score. A higher score indicates a better health state [20].

FAC thickness was measured by a board-certified physiatrist by using real-time ultrasonography (Zonare Medical, Co, Ltd., South Korea). The ultrasonography was set at a frequency of 14 MHz and a depth of 25 mm. We drew three vertical parallel lines on a transparent sheet placed against the screen of the ultrasonography machine, with one line at the center of the screen and two lines midway between the center and the lateral edges of the screen. The affected knee was flexed maximally in a supine position. The transducer was allocated transversely to the leg just above the superior margin of the patella and perpendicularly to the bone surface to optimize the FAC. The midpoint of the intercondylar notch was imaged at the center of the machine screen. FAC thickness was measured perpendicular to the bone-cartilage interface at three areas, which were the three lines drawn on the transparent sheets intersecting the bone-cartilage interface at the medial condyle, intercondylar notch, and lateral condyle [21].

### 2.5. Statistical Analysis

Statistical analysis was performed using SSPS version 18 for Windows (SSPS Inc., Chicago, IL, USA). Baseline descriptive statistics were compared using independent *t*-test for continuous data and Fisher's exact test for categorical data. Repeated measures analysis of variance (RM-ANOVA) was used to reveal the interactions between time and the groups. Statistical significance was defined as *P* > 0.05. Post hoc analysis was performed using independent *t*-test to compare values between the groups. Bonferroni correction was applied to adjust the two time period comparisons. Therefore, *P* > 0.025 was considered statistically significant for the post hoc analyses.

## 3. Results

### 3.1. General Characteristics of the Subjects

**Figure 2:**
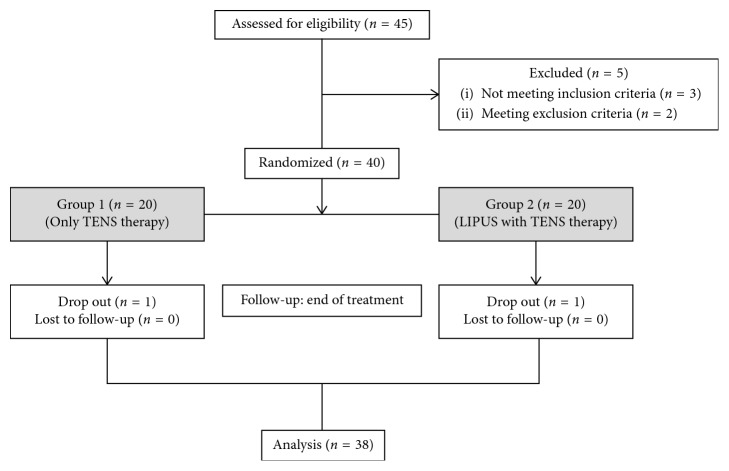
Flow chart of the study.

Forty participants (32 women and 8 men) aged 45–85 years (mean age ± SD, 57.60 ± 8.26 years) were recruited between November 10, 2017, and August 16, 2018. Twenty patients were randomly allocated to each group. Two patients dropped out due to a history of steroid injection into the affected knee or initiation of NSAIDs during the trial. Therefore, 19 patients from each group completed the treatment and evaluation (Figure 2).

Demographic data and baseline characteristics of each group are shown in Table 1. In the only TENS and LIPUS with TENS groups, the mean ages of the subjects were 56.00 ± 7.43 and 59.47 ± 9.20 years (*P*=0.209) and the mean durations of knee pain (months) were 62.74 ± 65.58 and 64.84 ± 62.70, respectively (*P*=0.803). The sex ratios (male : female) were 5 : 14 and 3 : 16, respectively. The significance of sex distribution for the group was 0.693. In the only TENS group, nine participants had K-L grade 1, eight had K-L grade 2, two had K-L grade 3, and none had K-L grade 4. In the LIPUS with the TENS group, six participants had K-L grade 1, nine had K-L grade 2, three had K-L grade 3, and one had K-L grade 4 (*P*=0.602).

Thus, no significant difference was found in age, sex, duration of knee pain, K-L grade, VAS, SF-36, WOMAC index scores, and FAC thickness at pretreatment between the two groups.

### 3.2. Comparison of Outcomes between the Groups

RM-ANOVA for the comparison of outcomes between groups revealed a significant time by group interaction for VAS-P2 (*P*=0.022), VAS-P3 (*P*=0.047), and WOMAC physical function score (*P*=0.026). No significant time by group interaction was found for VAS-P1, WOMAC pain score, WOMAC stiffness score, WOMAC total score, FAC thickness, and SF-36 global score (Table 2).

However, significant differences of VAS-P2, VAS-P3, and WOMAC physical function score were not noted between groups in the post hoc test. No significant differences between the groups for changes from baseline were found (Table 3).

### 3.3. Comparison between Pretreatment and Posttreatment Outcomes within the Groups

The results of the RM-ANOVA for the comparison between pretreatment and posttreatment outcomes within the group are shown in Table 4. VAS-P1 (*P* < 0.001), VAS-P2 (*P* < 0.001), VAS-P3 (*P* < 0.001), WOMAC pain score (*P* < 0.001), WOMAC physical function score (*P* < 0.001), WOMAC total score (*P* < 0.001), and SF-36 global score (only TENS group, *P*=0.004; LIPUS with TENS group, *P*=0.001) showed statistical differences over time in both groups. WOMAC stiffness score (*P* < 0.001) showed a statistical difference over time only in the LIPUS with TENS group. FAC thickness showed no significant differences (Table 4).

### 3.4. Adverse Effects

Adverse effects from the treatment were not observed.

## 4. Discussion

In this study, we aimed to elucidate the effects and safety of a stimulator using LIPUS combined with TENS on pain relief, functional improvement, and cartilage repair in patients with painful knee OA. Moreover, we aimed to prove the superiority of the effects of LIPUS combined with TENS therapy compared with only TENS therapy, which is widely used in the clinical field.

This study showed no significant difference in the treatment effect between the two groups. In the comparison of pretreatment and posttreatment outcomes within the groups, VAS, WOMAC pain score, WOMAC physical function score, WOMAC total score, and SF-36 global score revealed statistical differences in both groups. However, the WOMAC stiffness score showed a statistical difference only in the LIPUS with TENS group after treatment. No significant difference was noted in FAC thickness after treatment in either group.

The American College of Rheumatology strongly recommends exercise and weight loss for nonpharmacologic therapies of painful knee OA. In addition, it conditionally recommends manual therapy in combination with supervised exercise, thermal agent, and TENS [8]. The Osteoarthritis Research Society International guidelines advocate the use of biomechanical interventions and intra-articular corticosteroid injection, including exercise and weight management, as appropriate treatment modality for painful knee OA [9].

The current systematic review is inconclusive about the efficacy of TENS for the treatment of knee OA because of poor methodological quality and high degree of heterogeneity among the trials [11]. However, numerous randomized controlled studies have been conducted about the efficacy of TENS for painful knee OA, and those modalities have been widely used in the clinical setting [22–26]. Although TENS is not recommended to acute pain that is caused by infection or active bleeding tissue, there was tentative evidence that TENS reduces acute pain that is associated with postoperation, physical trauma, and medical procedure [16, 27–29]. TENS is based on the gate-control theory. This suggests that the stimulation of large diameter and primary sensory afferent cutaneous fibers, such as A-beta, activates inhibitory interneurons in the spinal cord dorsal horn, which leads to the inhibition of nociceptive signal transmission from small-diameter A-delta and C fibers [11, 30].

The current systematic review suggested a possible beneficial effect of therapeutic ultrasound for knee OA despite the low quality of the evidence [13]. Therapeutic ultrasound is based on the application emitting high-frequency sound waves to the tissues to obtain mechanical or thermal effects [13]. Therapeutic ultrasound delivery can be continuous or pulsed, and it can be divided into low-intensity (0.125–3 W/cm^2^) ultrasound or high-intensity (>5 W/cm^2^) ultrasound [31]. High-intensity ultrasound produces thermal effects with heating and causes an increase in metabolic activity and blood flow. It can also generate analgesic effects on nerve and contribute to pain relief [32].

LIPUS produces nonthermal effects, such as stable cavitation and acoustic microstreaming, which could alter membrane permeability and stimulate cell activity. These alteration and stimulation lead to increased protein synthesis, mast cell degranulation, growth factor production, calcium uptake, and fibroblast mobility, which all contribute to soft tissue healing [33, 34]. There are some animal studies about not only soft tissue healing but also cartilage repair [35, 36]. However, human studies about the cartilage-repair effect of LIPUS are scarce. Definite recommendations on the dose of energy, intensity, mode, and application technique of LIPUS required for cartilage repair have also not been established [12].

We used CARESTAR (GENEMEDI Co, Ltd., South Korea) to perform LIPUS combined with TENS therapy. This device gives LIPUS energy and TENS in 1-s shifts, and it is expected to create a synergistic effect of pain relief by TENS and cartilage repair by LIPUS. Therefore, we expected that LIPUS combined with TENS therapy would be superior to only TENS therapy. However, the combination therapy was not significantly superior to only TENS therapy. There was no statistically significant difference between the two therapies. In the between-group comparison using RM ANOVA, a significant time by group interaction was noted for VAS-P2 (*P*=0.022), VAS-P3 (*P*=0.047), and WOMAC physical function score (*P*=0.026) (Table 2). However, no statistically significant difference of the between-group comparison was found for VAS P-2 (*P* value of ΔV1−V2 = 0.027, *P* value of ΔV1−V3 = 0.457), VAS-P3 (*P* value of ΔV1−V2 = 0.134, *P* value of ΔV1−V3 = 0.759), and WOMAC physical function score (*P* value of ΔV1−V2 = 0.048, *P* value of ΔV1−V3 = 0.668) for changes from baseline (post hoc test; Table 3).

In the within-group comparison, both treatments (only TENS therapy and LIPUS with TENS therapy) demonstrated statistical differences for pain and physical function from baseline values. VAS-P1 (*P* < 0.001), VAS-P2 (*P* < 0.001), VAS-P3 (*P* < 0.001), WOMAC pain score (*P* < 0.001), WOMAC physical function score (*P* < 0.001), WOMAC total score (*P* < 0.001), and SF-36 global score (only TENS group, *P*=0.004; LIPUS with TENS group, *P*=0.001) showed statistical differences over time in both groups. The WOMAC stiffness scores (*P* < 0.001) showed a statistical difference over time only in the LIPUS with TENS group. Moreover, FAC thickness showed no significant differences after treatment in either group (Table 4). Although there was a statistical difference between pretreatment and posttreatment outcomes within the groups, it is difficult to determine if those differences are due to a therapeutic effect. This is because we did not have a sham (or no treatment) group. It is possible that improvements in the VAS, WOMAC, and SF-36 global score were due to a placebo effect.

We measured the FAC thickness via real-time ultrasonography to prove whether the LIPUS with TENS therapy had an effect on cartilage regeneration. A previous study on human cadaver revealed that the signal of LIPUS can propagate within the joint space of the human knee [17]. We also applied LIPUS with TENS therapy on the affected knee flexed at 90° to maximize the propagation of LIPUS signal. Although the optimal amount of LIPUS signal for cartilage repair is unknown, data from a rabbit osteochondral defect model indicated that the duration of ultrasound therapy and the quality of tissue repair may be correlated [37]. Therefore, we designed that each participant could receive more treatment sessions (more than 80 sessions for two months) than a previous study [12, 35, 36]. However, no significant difference was found in the FAC thickness after treatment in both groups.

Previous studies with an animal model proved that LIPUS accelerates cartilage healing during acute phase by starting ultrasound treatment 24–48 hours after the induction of acute arthritis [35, 36]. The study about ultrasound treatment of fibular fracture in rats revealed that bone healing is promoted in the early proliferative phases of repair. However, exposure during the late proliferative phase shows disadvantages such as delay in bone union [38]. Although the mechanism for cartilage repair by LIPUS is not clearly elucidated, application of LIPUS at the early phase of OA seems to be important to promote cartilage healing. However, each participant in this study classified with K-L grade 1 or higher indicating OA has already progressed. We assumed that it is one of the reasons why there was no difference in cartilage thickness after treatment in this study.

Experiencing minor skin irritation or contact dermatitis beneath electrodes is possible even though serious adverse events from TENS are rare [16]. Adverse effects by LIPUS are also known to be rare [39]. No adverse events occurred in both the groups in this study.

This study has several limitations. First, there was no sham group. Thus, we could not determine if the differences between the pretreatment and posttreatment outcomes were due to a therapeutic or placebo effect. Further investigation with the use of a sham group is warranted. Second, only 40 participants were recruited; thus, more studies with larger sample size and longer follow-up periods are required. Third, treatments were conducted as home-based self-therapy, but the participants completed a self-therapy checklist daily. Moreover, they were contacted by phone once a week and visited at home once a month to monitor the home-based self-therapy. Finally, we could not investigate the histologic changes to confirm the cartilage regeneration because it was a human clinical study.

## 5. Conclusions

The effects of a stimulator using LIPUS with TENS on pain relief and functional improvement were not superior to the only TENS therapy. Cartilage regeneration, which was expected as an additional benefit of LIPUS, was also not evident. Therefore, further investigation is warranted to prove whether the combination therapy has benefits over the use of only TENS therapy.

## Figures and Tables

**Table 1:** Baseline characteristics.

Variables	Only TENS group (*n* = 19)	LIPUS with TENS group (*n* = 19)	*P* value
Age (year)	56.00 ± 7.43	59.47 ± 9.20	0.209
Sex (male: female)	5 : 14	3 : 16	0.693
Duration of pain (months)	62.74 ± 65.58	64.84 ± 62.70	0.803
Kellgren–Lawrence score (*n*)			
Grade I	9	6	0.602
Grade II	8	9	
Grade III	2	3	
Grade IV	0	1	
VAS-P1	4.00 ± 1.37	4.16 ± 1.50	0.752
VAS-P2	4.95 ± 1.51	4.89 ± 1.24	0.928
VAS-P3	2.68 ± 1.34	3.00 ± 1.15	0.441
WOMAC			
Pain	7.53 ± 3.67	8.63 ± 3.09	0.322
Stiffness	1.26 ± 1.79	1.53 ± 1.50	0.418
Physical function	20.89 ± 11.79	25.05 ± 11.20	0.273
Total score	29.68 ± 15.83	35.21 ± 14.74	0.307
FAC thickness (cm)			
Medial	0.20 ± 0.03	0.20 ± 0.03	0.751
Central	0.21 ± 0.04	0.20 ± 0.04	0.325
Lateral	0.20 ± 0.03	0.20 ± 0.03	0.908
SF-36 global score	58.25 ± 17.08	59.11 ± 16.59	0.651

Data are presented as mean ± SD. ^*∗*^*P* < 0.05. TENS, transcutaneous electrical nerve stimulation; LIPUS, low-intensity pulsed ultrasound; VAS, visual analogue scale (range, 1 to 10), with higher values indicating worse outcomes; VAS-P1, visual analogue scale for knee pain at the current moment; VAS-P2, visual analogue scale for knee pain with movement; VAS-P3, visual analogue scale for knee pain at the resting position; WOMAC, Western Ontario and McMaster index with higher scores representing worse pain, stiffness, and impaired physical function; FAC, femoral articular cartilage; SF-36, the MOS 36-Item Short-Form Health Survey with a higher score indicating better health.

**Table 2:** Comparison of outcomes between the groups.

Variables	V1	V2	V3	Time X group *P* value
VAS-P1				
Only TENS group	4.00 (0.32)	2.47 (0.36)	2.63 (0.43)	0.080
LIPUS + TENS group	4.16 (0.34)	1.89 (0.21)	2.53 (0.29)	
VAS-P2				
Only TENS group	4.95 (0.35)	3.16 (0.41)	3.00 (0.45)	0.022^*∗*^
LIPUS + TENS group	4.89 (0.29)	2.21 (0.18)	2.74 (0.20)	
VAS-P3				
Only TENS group	2.68 (0.31)	1.32 (0.39)	1.05 (0.36)	0.047^*∗*^
LIPUS + TENS group	3.00 (0.26)	1.05 (0.19)	1.53 (0.23)	
WOMAC				
Pain				
Only TENS group	7.53 (0.84)	4.63 (0.84)	4.26 (0.86)	0.160
LIPUS + TENS group	8.63 (0.71)	4.53 (0.58)	5.32 (0.71)	
Stiffness				
Only TENS group	1.26 (0.41)	0.58 (0.29)	0.74 (0.35)	0.298
LIPUS + TENS group	1.53 (0.35)	0.68 (0.25)	0.58 (0.19)	
Physical function				
Only TENS group	20.89 (2.71)	13.84 (2.38)	10.79 (2.21)	0.026^*∗*^
LIPUS + TENS group	25.05 (2.57)	13.89 (1.99)	15.84 (2.31)	
Total				
Only TENS group	29.68 (3.63)	19.05 (3.30)	15.79 (3.22)	0.055
LIPUS + TENS group	35.21 (3.38)	19.11 (2.67)	21.74 (3.03)	
FAC thickness (cm)				
Medial				
Only TENS group	0.20 (0.01)	0.20 (0.01)	0.21 (0.01)	0.453
LIPUS + TENS group	0.20 (0.01)	0.20 (0.01)	0.20 (0.01)	
Central				
Only TENS group	0.21 (0.01)	0.21 (0.01)	0.21 (0.01)	0.635
LIPUS + TENS group	0.20 (0.01)	0.20 (0.01)	0.20 (0.01)	
Lateral				
Only TENS group	0.20 (0.01)	0.20 (0.01)	0.20 (0.01)	0.666
LIPUS + TENS group	0.20 (0.01)	0.20 (0.01)	0.20 (0.01)	
SF-36 global score				
Only TENS group	58.25 (3.92)	66.34 (3.72)	67.34 (4.18)	0.659
LIPUS + TENS group	59.11 (3.81)	69.85 (2.73)	67.80 (2.53)	

Data are presented as mean (SE). ^*∗*^*P* < 0.05. V1, visit 1 (pretreatment); V2, visit 2 (posttreatment period 1); V3, visit 3 (posttreatment period 2); TENS, transcutaneous electrical nerve stimulation; LIPUS, low-intensity pulsed ultrasound; VAS, visual analogue scale (range, 1 to 10), with higher values indicating worse outcomes; VAS-P1, visual analogue scale for knee pain at the current moment; VAS-P2, visual analogue scale for knee pain with movement; VAS-P3, visual analogue scale for knee pain at the resting position; WOMAC, Western Ontario and McMaster index with higher scores representing worse pain, stiffness, and impaired physical function; FAC, femoral articular cartilage; SF-36, the MOS 36-Item Short-Form Health Survey with a higher score indicating better health.

**Table 3:** Differences between the groups for changes from baseline.

Variables	ΔV1−V2	Difference between groups in change from baseline (97.5% CI)	*P* value	ΔV1−V3	Difference between groups in change from baseline (97.5% CI)	*P* value
VAS-P1						
Only TENS group	1.53 (0.34)	−0.74 (−1.70 to 0.23)	0.036	1.37 (0.41)	−0.26 (−1.61 to 1.08)	0.650
LIPUS + TENS group	2.26 (0.24)			1.63 (0.40)		
VAS-P2						
Only TENS group	1.79 (0.35)	−0.89 (−2.01 to 0.22)	0.027	1.95 (0.42)	−0.21 (−1.50 to 1.08)	0.457
LIPUS + TENS group	2.68 (0.32)			2.16 (0.36)		
VAS-P3						
Only TENS group	1.37 (0.28)	−0.58 (−1.47 to 0.31)	0.134	1.63 (0.31)	0.16 (−1.04 to 1.35)	0.759
LIPUS + TENS group	1.95 (0.26)			1.47 (0.41)		
WOMAC						
Pain						
Only TENS group	2.89 (0.55)	−1.21 (−3.23 to 0.81)	0.170	3.26 (0.59)	−0.05 (−2.49 to 2.39)	0.960
LIPUS + TENS group	4.11 (0.67)			3.32 (0.86)		
Stiffness						
Only TENS group	0.68 (0.31)	−0.16 (−1.06 to 0.74)	0.653	0.53 (0.29)	−0.42 (−1.31 to 0.47)	0.241
LIPUS + TENS group	0.84 (0.23)			0.95 (0.25)		
Physical function						
Only TENS group	7.05 (1.39)	−4.11 (−8.68 to 0.47)	0.048	10.11 (1.55)	0.89 (−3.95 to 5.74)	0.668
LIPUS + TENS group	11.16 (1.38)			9.21 (1.37)		
Total						
Only TENS group	10.6 (1.82)	−5.47 (−11.78 to 0.83)	0.050	13.89 (1.97)	0.42 (−6.42 to 7.26)	0.886
LIPUS + TENS group	16.11 (1.99)			13.47 (2.16)		
FAC thickness (cm)						
Medial						
Only TENS group	0.00 (0.00)	0.00 (−0.00 to 0.00)	0.623	−0.01 (0.00)	−0.00 (−0.02 to 0.01)	0.931
LIPUS + TENS group	0.00 (0.00)			−0.01 (0.00)		
Central						
Only TENS group	0.00 (0.00)	0.00 (−0.00 to 0.00)	0.636	0.00 (0.00)	−0.00 (−0.01 to 0.01)	0.808
LIPUS + TENS group	0.00 (0.00)			0.00 (0.00)		
Lateral						
Only TENS group	0.00 (0.00)	0.00 (−0.00 to 0.01)	0.695	0.00 (0.00)	−0.00 (−0.01 to 0.01)	0.483
LIPUS + TENS group	0.00 (0.00)			0.00 (0.00)		
SF-36 global score						
Only TENS group	−8.09 (2.28)	2.65 (−6.08 to 11.38)	0.328	−9.09 (3.14)	−0.41 (−10.37 to 9.56)	0.925
LIPUS + TENS group	−10.74 (2.95)			−8.69 (2.87)		

Data are presented as mean (SE). ^*∗*^*P* < 0.025. Bonferroni correction was applied to adjust the 2-time comparisons. ΔV1−V2, difference of values between visit 1 and visit 2; ΔV1−V3, difference of values between visit 1 and visit 3; CI; confidence interval; TENS, transcutaneous electrical nerve stimulation; LIPUS, low-intensity pulsed ultrasound; VAS, visual analogue scale (range, 1 to 10), with higher values indicating worse outcomes; VAS-P1, visual analogue scale for knee pain at the current moment; VAS-P2, visual analogue scale for knee pain with movement; VAS-P3, visual analogue scale for knee pain at the resting position; WOMAC, Western Ontario and McMaster index with higher scores representing worse pain, stiffness, and impaired physical function; FAC, femoral articular cartilage; SF-36, the MOS 36-Item Short-Form Health Survey with a higher score indicating better health.

**Table 4:** Comparison of pretreatment and posttreatment outcomes within the groups.

Variables	V1	V2	V3	*P* value (time)
VAS-P1				
Only TENS group	4.00 (0.32)	2.47 (0.36)	2.63 (0.43)	<0.001^*∗*^
LIPUS + TENS group	4.16 (0.34)	1.89 (0.21)	2.53 (0.29)	<0.001^*∗*^
VAS-P2				
Only TENS group	4.95 (0.35)	3.16 (0.41)	3.00 (0.45)	<0.001^*∗*^
LIPUS + TENS group	4.89 (0.29)	2.21 (0.18)	2.74 (0.20)	<0.001^*∗*^
VAS-P3				
Only TENS group	2.68 (0.31)	1.32 (0.39)	1.05 (0.36)	<0.001^*∗*^
LIPUS + TENS group	3.00 (0.26)	1.05 (0.19)	1.53 (0.23)	<0.001^*∗*^
WOMAC				
Pain				
Only TENS group	7.53 (0.84)	4.63 (0.84)	4.26 (0.86)	<0.001^*∗*^
LIPUS + TENS group	8.63 (0.71)	4.53 (0.58)	5.32 (0.71)	<0.001^*∗*^
Stiffness				
Only TENS group	1.26 (0.41)	0.58 (0.29)	0.74 (0.35)	0.106
LIPUS + TENS group	1.53 (0.35)	0.68 (0.25)	0.58 (0.19)	<0.001^*∗*^
Physical function				
Only TENS group	20.89 (2.71)	13.84 (2.38)	10.79 (2.21)	<0.001^*∗*^
LIPUS + TENS group	25.05 (2.57)	13.89 (1.99)	15.84 (2.31)	<0.001^*∗*^
Total				
Only TENS group	29.68 (3.63)	19.05 (3.30)	15.79 (3.22)	<0.001^*∗*^
LIPUS + TENS group	35.21 (3.38)	19.11 (2.67)	21.74 (3.03)	<0.001^*∗*^
FAC thickness (cm)				
Medial				
Only TENS group	0.20 (0.01)	0.20 (0.01)	0.21 (0.01)	0.073
LIPUS + TENS group	0.20 (0.01)	0.20 (0.01)	0.20 (0.01)	0.134
Central				
Only TENS group	0.21 (0.01)	0.21 (0.01)	0.21 (0.01)	0.355
LIPUS + TENS group	0.20 (0.01)	0.20 (0.01)	0.20 (0.01)	0.162
Lateral				
Only TENS group	0.20 (0.01)	0.20 (0.01)	0.20 (0.01)	0.141
LIPUS + TENS group	0.20 (0.01)	0.20 (0.01)	0.20 (0.01)	0.162
SF-36 global score				
Only TENS group	58.25 (3.92)	66.34 (3.72)	67.34 (4.18)	0.004^*∗*^
LIPUS + TENS group	59.11 (3.81)	69.85 (2.73)	67.80 (2.53)	0.001^*∗*^

Data are presented as mean (SE). ^*∗*^*P* < 0.05.

## Data Availability

The data used to support the findings of this study are available from the corresponding author upon request.
